# Exploring Viral Genome Profile in Mpox Patients during the 2022 Outbreak, in a North-Eastern Centre of Italy

**DOI:** 10.3390/v16050726

**Published:** 2024-05-03

**Authors:** Michela Deiana, Denise Lavezzari, Antonio Mori, Silvia Accordini, Elena Pomari, Chiara Piubelli, Simone Malagò, Maddalena Cordioli, Niccolò Ronzoni, Andrea Angheben, Evelina Tacconelli, Maria Rosaria Capobianchi, Federico Giovanni Gobbi, Concetta Castilletti

**Affiliations:** 1 Department of Infectious-Tropical Diseases and Microbiology, IRCCS Sacro Cuore Don Calabria Hospital, Negrar di Valpolicella, 37024 Verona, Italymrcapobianchi@gmail.com (M.R.C.); federico.gobbi@sacrocuore.it (F.G.G.); concetta.castilletti@sacrocuore.it (C.C.); 2 PhD National Programme in One Health approaches to infectious diseases and life science research, Department of Public Health, Experimental and Forensic Medicine, University of Pavia, 27100 Pavia, Italy; 3 Division of Infectious Diseases, Department of Diagnostic and Public Health, University of Verona, 37134 Verona, Italy; 4 Division of Infectious Diseases, Department of Medicine, Verona University Hospital, 37134 Verona, Italy; evelina.tacconelli@univr.it; 5 Department of Clinical and Experimental Sciences, University of Brescia, 25121 Brescia, Italy

**Keywords:** monkeypox virus (MPXV), mpox outbreak, next-generation sequencing (NGS), mutations, APOBEC3 intra-patient variation, single nucleotide variant (SNV)

## Abstract

In 2022, an unprecedented outbreak of mpox raged in several nations. Sequences from the 2022 outbreak reveal a higher nucleotide substitution if compared with the estimated rate for orthopoxviruses. Recently, intra-lesion SNVs (single nucleotide variants) have been described, and these have been suggested as possible sources of genetic variation. Until now, it has not been clear if the presence of several SNVs could represents the result of local mutagenesis or a possible co-infection. We investigated the significance of SNVs through whole-genome sequencing analysis of four unrelated mpox cases. In addition to the known mutations harboured by the circulating strains of virus (MPXV), 7 novel mutations were identified, including SNVs located in genes that are involved in immune evasion mechanisms and/or viral fitness, six of these appeared to be APOBEC3-driven. Interestingly, three patients exhibited the coexistence of mutated and wild-type alleles for five non-synonymous variants. In addition, two patients, apparently unrelated, showed an analogous pattern for two novel mutations, albeit with divergent frequencies. The coexistence of mixed viral populations, harbouring non-synonymous mutations in patients, supports the hypothesis of possible co-infection. Additional investigations of larger clinical cohorts are essential to validating intra-patient viral genome heterogeneity and determining the possibility of co-presence events of slightly divergent MPXV strains.

## 1. Introduction

Monkeypox virus is a zoonotic enveloped double-stranded DNA Orthopoxvirus [1]. It was discovered in 1958 after an outbreak of a smallpox-like disease in macaques [1]. Several animals can be infected with MPXV, and their most probable animal reservoir is found in all arboreal rodents [2]. Recently, human-to-human transmission has been reported, driven by close contact with the body fluids, skin lesions, and respiratory droplets of infected individuals [1]. Since May 2022, several cases of mpox have been reported in non-endemic countries and the virus has spread globally [1]. The first confirmed cases in Europe had no apparent link to endemic countries. Instead, patients shared a history of travel to Portugal and the Canary Islands, as well as sexual behavior (men who have sex with men, MSM), and the majority were aged between 20 and 50 years [1]. The infection rapidly spread worldwide, and the WHO announced a Public Health Emergency of International Concern (PHEIC) due to MPXV spread worldwide [3]. As of 11 December 2023, there were 92,167 confirmed cases of mpox and 170 deaths were reported across 117 countries [4]. Since 2022, a total of 7530 genome sequences have been reported in the GISAID (https://gisaid.org/, last accessed 20 December 2023) database, including 31 sequences derived from Italian cases. After peaking in July 2022, the epidemic curve gradually declined, and the end of the PHEIC state was declared on 11 May 2023. The MPXV genome is 197 Kb long, harboring approximately 190 genes [5]. The genome’s architecture is divided into three different regions: the highly conserved core region, which encodes for essential replication and assembly proteins [6], and the right and left variable regions, whose activity mainly relates to pathogenic factors [5]. The non-coding regions and ITRs are additional genomic structures that are potentially involved in MPVX evolution during an outbreak [7]. Phylogenetic characterization embraces two clades: the Central African Clade or Clade I, and the West African Clade or Clade IIa and b. [8,9]. Circulating strains from the most recent outbreak fell into the B.1 lineage [9,10]. According to the available sequences, the MPXV that caused the 2022 outbreak was phylogenetically related to the viral strains that caused the 2017–2018 outbreak in West Africa, and it evolved through point mutation accumulation [10,11]. Overall, 129 viral genomes, sharing ~50 SNVs (single nucleotide variants) and differing from the sequences of the 2018–2019 MPXV, have been shared through GISAID. As reported in the literature [12], the number of SNVs in the sequences from the 2022 outbreak appears to be higher than the estimated substitution rate for Orthopoxviruses (i.e., 1–2 substitutions per site per year) [13]. Most changes involved extensive G-to-A/C-to-T replacements, suggesting that the intrinsic host antiviral mechanism, based on APOBEC3 enzymes, may have driven MPXV evolution since 2017 [8,14,15]. Mutations seems to preferentially affect highly expressed viral genes, possibly because ongoing transcription exposes single-stranded DNA stretches, making them targets of APOBEC3 editing [16,17]. In the present study, we performed whole-genome analysis of viral sequences, extracted from patients diagnosed with the mpox disease, in order to investigate the evolution of MPXV in Italy.

## 2. Materials and Methods

### 2.1. Clinical Samples

The collected clinical samples were originally sent for diagnostic purposes to the IRCCS Sacro Cuore Don Calabria Hospital referral regional laboratory, located in Negrar di Valpolicella (Verona, Veneto Region, Italy). Skin lesion swabs of patients presenting mpox clinical manifestations, i.e., testing positive for MPXV during qPCR and showing a Ct value ≤ 20 (RealStar^®^ Orthopoxvirus PCR Kit 1.0 RUO, Altona, Hamburg, Germany), were used to perform full-genome sequencing analysis. Thus, a total of four samples (P1, P2, P3 and P4) were selected; all of them were collected from male patients. Specifically, P1 and P2 dated back to the early phase (June–July 2022) of the Italian epidemic curve, while P3 and P4 were traced to the late phase (October–November 2022). Three out four patients had been travelling (P1, Balearic Islands; P2, Spain; P4, Bosnia and Herzegovina). P1, P2, and P3 declared sexual risk behaviors. The patients were unrelated to each other; P1 and P2 were HIV-positive. No other immune system disorders were reported that could affect the course of the disease. Clinical evolution was comparable in all four patients; no particular or different symptoms were reported. Laboratory diagnosis was also confirmed by using a multiplex real-time PCR assay for the qualitative detection of both MPXV and OPXV nucleic acids (STANDARD™ M10 MPX/OPX, SD Biosensor, Yeongtong-gu, Republic of Korea). Patients’ general information is reported in Supplementary Materials, Table S2.

### 2.2. DNA Extraction, Library Preparation and NGS

Working according to the manufacturer’s instructions, viral DNA was extracted from 200 µL of skin lesion swab medium and eluted in 60 µL using an EZ1 Advanced XL instrument and EZ1 DSP Virus Kit (Qiagen, Hilden, Germany).

DNA concentration was determined using the Qubit dsDNA HS assay kit (Thermofisher, Waltham, MA, USA) before whole-genome sequencing library preparation. For this purpose, the Ion Xpress™ Plus Fragment Library Kit (Thermofisher) was used, starting from 150 ng of gDNA. The quality of libraries was assessed by applying a D1000 ScreenTape kit to the 4200 TapeStation System (Agilent, Santa Clara, CA, USA). Single-read sequencing was performed by using the Ion 530™ Chip on the Ion S5™ instrument kit (Thermofisher) with a 550 flow.

### 2.3. Bioinformatics Analysis

Single-end reads were subjected to different bioinformatic procedures:

- Quality control (QC), performed using FastQC v.0.11.9;
- Adapter removal, performed using Fastp v.0.23.2;
- Contamination control, performed using Kraken2 v. 2.1.2;
- Alignment of the reads to the MPXV reference genome (NC_063383.1), performed using BWA-mem2 v. 2.2.1;
- Genome assembly, performed using iVar consensus v.1.3.1 tool (options “-aa -A -d o -Q 0”).

For variant determination and phylogenetic analysis, a multifasta file containing all GISAID sequences and our four assembled sequences was created and submitted to Nextclade web 2.11.0 (https://docs.nextstrain.org/projects/nextclade/en/stable/, last accessed 10 January 2024), using NC_063383.1 as a reference. Multiple alignments were performed on the Augur tool, a specific phylogenetic software designed for human pathogens that uses the maximum likelihood approach [18], using the default parameter settings. The resulting output file (in Newick format) was used to build the phylogenetic tree using iTOL (https://itol.embl.de/itol_account.cgi, last accessed 24 January 2024).

The identified variants were used as inputs for the “Mutation-Profile tool” in order to identify APOBEC3-derived variants (https://github.com/insapathogenomics/mutation_profile, accessed 20 November 2023). The SNVs identified by Nextclade were analysed using an in-house Python script. The variants identified during the variant calling, —both the new variants present exclusively in our sample and those known and reported in other studies—were analysed in order to understand their evolution over the years and their spread in the world by comparing them to MPXV genome sequences deposited in GISAID.

### 2.4. GISAID Data Download

A total of 4749 (up to 5 December 2023) complete [>196 kbs] MPXV sequences were downloaded from the GISAID EpiPox (https://gisaid.org/, accessed 24 January 2024) repository [19], excluding those that registered as low-coverage [>5% Ns], and these were used for comparison with our assembled sequences, as described above.

### 2.5. Sanger Sequencing

PCR and Sanger sequencing were performed to confirm the novel SNVs detected by NGS. Primers (Supplementary Material, Section S1) were designed using OligoPerfect software (Thermofisher, https://www.thermofisher.com/account-center/simplified-username.html, last accessed 25 November 2023). In order to confirm the specificity of primers, we also tested them on human genomic DNA.

The PCR protocol is described in Section S2 of the Supplementary Materials. PCR products were purified using ExoSAP-IT (Thermofisher) and sequenced using the BigDye Terminator v3.1 Cycle Sequencing Kit according to the manufacturer’s instructions and the ABI 3500 Genetic Analyzer (Thermofisher). Forward and reverse single spectra were manually inspected, and sequences analysed with Clustal Omega and Blast tools.

### 2.6. Ethics

The study protocol received ethical clearance from the Ethical Committee of Verona and Rovigo provinces (Prot. n. 6232 del 30 January 2023). Participants signed an informed con-sent form. Samples were stored in the “Tropica Biobank” of the IRCCS Sacro Cuore Don Calabria Hospital.

## 3. Results

### 3.1. QC Analyses, Read Alignment and Consensus Generation

Among the four sequenced samples (P1–P4), three (P1, P2 and P4) achieved a number of sequenced fragments between 16.4 Mb and 23.8 Mb, whereas only 2.5 Mb were obtained from P3. Alignment details are summarized in Supplementary Materials Section S3, Table S3. Sequences that passed the QC analyses (P1, P2 and P4) were then uploaded on GISAID and the corresponding EPID was reported in Table S4.

### 3.2. Variant Calling and Annotation

The consensus sequences from the four patients were compared with 4749 sequences downloaded from GISAID. They were then analysed in order to identify nucleotide mutations, substitutions, insertions, and deletions. Table 1A shows details relative to the novel mutations, while the complete list of mutations is available in Supplementary Material Table S1.

**Figure 1:**
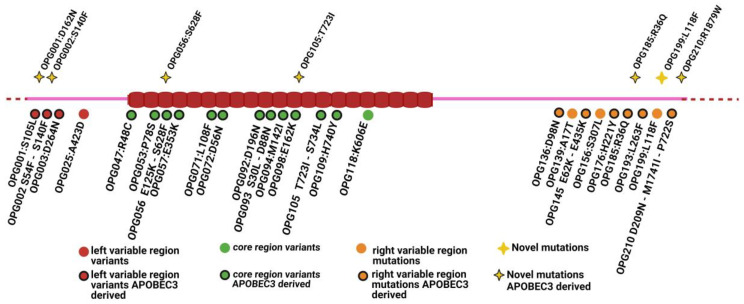
Genomic localization of the 36 mutations (known and novel) identified in the viral genome of the samples collected from the four patients. The brick-coloured beads represent the core region, while purple lines represent the left and right variable regions. The dotted line represents the ITR. Of the mutations, 13 are distributed in the core region (OPG047, OPG053, OPG056, OPG057, OPG071, OPG072, OPG092, OPG093, OPG094, OPG098, OPG105, OPG109, and OPG118), 9 in the right variable region (OPG136, OPG139, OPG145, OPG150, OPG156, OPG176, OPG185, OPG193, OPG199, OPG210), and 4 within the left variable region (OPG001, OPG002, OPG003, OPG025).

A total of 36 amino acid mutations were identified across 27 ORFs. Overall, 7 out of 36 mutations were novel and these were distributed across the whole MPXV genome (Figure 1); of these, 6 were predicted to be APOBEC3-derived.

Overall, 29 out of 36 mutations were already recorded in GISAID among the 2022 outbreak sequences and several had also been recorded in 2021, as shown in Supplementary Materials, Figure S1.

As expected, the geographical distribution of amino acid substitutions showed major diffusion in the European continent, as shown in Figure S2 in Supplementary Materials. Very few substitutions appeared to be shared with samples from the African continent, but this was likely due to bias, given the small number of sequences available from the latter.

Five out of the seven novel mutations located on the genes (OPG001, OPG002, OPG056, OPG105, OPG185, OPG199, and OPG210—shown in Table 1A), revealed a mixed profile (OPG001:D162N, OPG002:S140F, OPG185:R36Q, OPG199:L118F, and OPG210:R1879W), with some carrying the SNVs and others carrying wild-type reference nucleotides. The details of this are available in Supplementary Material, Section S4.

### 3.3. APOBEC3-Driven Mutations

The presence of nucleotide mutations due to the deaminase editing of APOBEC3 [20] was investigated among all identified SNVs. The details of this are available in Supplementary Materials, Table S5. Overall, analysis performed with “mutation profile tool” highlighted that 59 SNVs were located in the coding regions, resulting in 23 synonymous and 36 non-synonymous mutations. Among these, six out of the seven newly identified mutations appeared to be APOBEC3-driven, namely OPG001:D162N, OPG002:S140F, OPG056:S628F, OPG105:T723I, OPG185:R36Q, and OPG210:R1879W (Figure 1).

### 3.4. Phylogenetic Analysis

**Figure 2:**
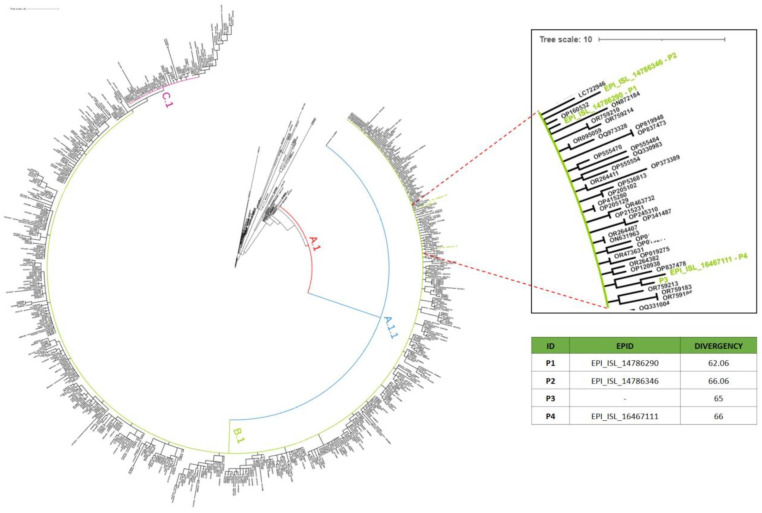
Phylogenetic analysis of MPXV sequences. The major subclades are represented with different colours (i.e., A in black, A.1 in red, A.1.1 in blue, B.1 in green, and C.1 in purple). Our four samples, all of which belong to Clade IIb, are highlighted in green (see magnified box on the right). The table shows mutation divergences of our four samples, compared with reference genome (NC_063383.1).

To investigate the single nucleotide differences in both imported and local cases, we calculated nucleotide distances between our four sequences and the whole-genome sequences of MPXV obtained from GISAID (Figure 2). Like the majority of sequences detected in Europe, the four sequences analysed in our study belonged to clade IIb, subclade B.1. The divergency data were reported in the phylogenetic tree, as shown in Figure 2.

### 3.5. APOBEC3-Driven Mutations

To confirm the co-presence of SNVs and wild-type alleles, the variants showing two different allele frequencies (OPG185:R36Q, OPG199:L118F, OPG210:R1879W) were inspected through Sanger sequencing. The mutations OPG001:D162N and OPG002:S140F were not confirmed with Sanger sequencing because they mapped on ITR regions; moreover, they showed a low mapping quality score due to the intrinsic non-univocity of ITRs. Amplicons were run on agarose gel, the electropherograms of which are shown in Supplementary methods, Section S5. As a control, P1 was confirmed to be wild-type for the tested gene positions (OPG185, OPG199 and OPG210). As summarized in Table 1B, Sanger sequencing of OPG185:R36Q, identified in sample P2, confirmed the co-presence of the two variants. OPG199 and OPG210 were tested in P3 and P4 samples. Sanger analyses of OPG199 identified the mutation in P4, while P3 resulted wild-type alleles. The investigation of OPG210 highlighted the mutation in P4, whereas P3 showed a wild-type allele (data shown in Supplementary methods, Section S5).

## 4. Discussion

The 2022 mpox outbreak, as the SARS-CoV-2 pandemic, has drawn attention to viral transmission across species, as well as to adaptability, infectivity, and diffusion outside of endemic areas. The contribution of genomic analysis of SARS-CoV-2 strains, isolated from various geographical regions, was of paramount importance in fighting the pandemic. Genomic studies of MPXV diffusion are thus crucial to characterizing the different circulating strains, identifying evolutionary links, and predicting transmission patterns. Recently, the presence of minor intra-lesion SNVs has been described [21,22], suggesting a possible direct origin of the mutation within the lesion of the index patient of the transmission chain [6]. To the best of our knowledge, it is still unclear if the co-presence of different nucleotide in a single lesion could be due to the local action of APOBEC3 or originate from co-infection with different SNV-carrying populations [6].

In this study, four whole-genome MPXV sequences that had been derived from the skin lesions of infected patients were analysed. Our aim was to determine the mutational profile of the virus and identify any potential novel mutations that may have been acquired over time. The infections occurred during two distinct periods of the epidemic in Italy: early spring (June–July 2022) and late autumn (October–November 2022). It is worth noting that three out of four patients had been travelling to Spain, the Balearic Islands, and Bosnia and Herzegovina, highlighting the primary role of people’s mobility in the spread of the virus across borders and emphasizing the importance of continuous surveillance and research. In agreement with previous reports [7,8,9], our sequences belonged to the current outbreak strain, i.e., Clade IIb, sublineage B.1. A total of 36 mutations were identified, of which 29 were already known, while seven had never been reported before. Interestingly, four known SNVs, located in genes OPG003, OPG056, OPG105, and OPG210, had already been described as being specific to subclade B.1 [10], and most of them seemed to be APOBEC3-derived mutations [7,10,22]. Among the novel non-synonymous SNVs, three were located in OPG056, OPG105, and OPG210 genes, which are known to be prone to mutations [7]. As described elsewhere, OPG056 is responsible for the maturation of extracellular enveloped viral protein, which is important for the transportation of viral particles from intracellular compartments to cell membrane [11]. It could be speculated that the variations in this gene could represent a novel mechanism of immune escape. Similarly, we observed mutations in the OPG185 gene, which is associated with virulence factors and immune evasion [12], and in the OPG199 gene, which is known to be an inhibitor of apoptosis and of caspase 1 and 8 [23]. Proteins encoded by OPG105 and OPG210 have been proposed to play an indirect role in immunomodulation [10,14,15,16]. In our study, we also identified variants in the ITRs, which are considered to be hot-spots for mutations [17]. Intriguingly, these mutations predominantly affected proteins with functions related to the immune modulation of the host [17], emphasizing the intricate interplay between viral mutations and the host’s immune response. Among the novel SNVs, five were found to be co-expressed with the corresponding wild-type alleles. To confirm NGS results, we performed Sanger sequencing on selected genes that showed good-quality mapping (OPG185:R36Q, OPG199:L118F, OPG210:R1879W). Both OPG199:L118F and OPG210:R1879W were detected in two unrelated patients and there was an increasing frequency of the mutated allele over time. Sanger results confirmed the co-presence of the wt and the mutated allele of OPG185:R36Q in the P2 sample. For P4 samples, OPG199:L118F and OPG210:R1879W were found to be mutated, while P3 resulted in wt. Considering these results, it is important to highlight that the Sanger technique has lower sensitivity compared to NGS [7,10]. Nonetheless, our observation still endorses the coexistence of different populations in the same biological sample. Taken together, these findings lend credence to the hypothesis of the possible coexistence of different viral strains within the same lesion. However, further evidence is needed to prove whether our observation is due to real co-infection with different viral populations or is the result of the extended activity of APOBEC3, which has led to virus’ diversification and adaptation into diverging MPXV strains. In light of our findings, the presence of two different novel mutations in two apparently unrelated patients, infected within the same timeframe, lends weight to the co-infection and thus the co-circulation of different viral populations.

It is thus crucial to continue research in this area in order to fully understand the implications of these findings and their potential impact on future treatment strategies and public health measures.

## 5. Conclusions

Our analysis shows that MPXV has accumulated multiple different SNVs in genes, with functional implications for viral infection and transmission. Thus, it seems reasonable to speculate that the detected mutations may be associated with phylogenetic evolution, which may have facilitated the spread of the virus worldwide. Moreover, the simultaneous presence of different viral sub-populations, with very similar mutation patterns, in unrelated patients, suggests a real possibility of co-infection and co-circulation. More in-depth investigations, including the in vitro viral isolation of the different sub-populations as well as the investigation of different biological matrices, are needed to elucidate this point. Overall, genomic surveillance is required to confirm our hypothesis, to identify evolutionary constraints that guide MPXV mutation, and to monitor the emergence and spread of new strains that may eventually exert more aggressive pathogenic behavior.

## Figures and Tables

**Table 1:** (**A**) Characteristics of novel SNVs. The coverage depth of coverage (DOC) of the identified mutation, SNV annotation, gene/aa substitution, gene function, and read frequency of the mutated and wt alleles are reported. (**B**) Sanger sequencing validation of mixed-frequency SNV/wt alleles. Each line refers to one of the three analysed mutations. For each sample, the frequency of wild-type and mutated alleles was reported.

**(A)**											
**ID**	**EPID**		**D_o_C(X)**	**SNV**	**GENE/AA SUBs**		**GENE FUNCTION**			**READ FREQ**	
P4	EPI_ISL_16467111		91	C1092T	OPG001:D162N		Chemokine binding protein			78% mutated 22% wild-type	
P2	EPI_ISL_14786346		102	G2333A	OPG002:S140F		Crm-B-secreted TNF-alpha-receptor-like protein			72% mutated 28% wild-type	
P4	EPI_ISL_16467111		107	G37152A	OPG056:S628F		EEV maturation protein			100% mutated	
P3	-		23							100% mutated	
P1	EPI_ISL_14786290		45	C83293T	OPG105:T723I		DNA-dependent RNA polymerase subunit rpo147			100% mutated	
P2	EPI_ISL_14786346		70	G159023A	OPG185:R36Q		Hemagglutinin			77% mutated 23% wild-type	
P4	EPI_ISL_16467111		110	G171341T	OPG199:L118F		Serpin			80% mutated 20% wild-type	
P3	-		11							27% mutated 73% wild-type	
P4	EPI_ISL_16467111		92	C186990T	OPG210:R1879W		B22R family protein			80% mutated 20% wild-type	
P3	-		13							54% mutated 46% wild-type	
**(B)**											
		**NGS**						**SANGER**			
		P2		P3		P4		P2	P3		P4
OPG185:G159023A		77% mutated 23% wild-type						wild-type and mutated	-		-
OPG199:C171341T				27% mutated 73% wild-type		80% mutated 20% wild-type		-	wild-type		mutated
OPG210:C186990T				54% mutated 46% wild-type		80% mutated 20% wild-type		-	wild-type		mutated

## Data Availability

All the dataset containing demographic information and experimental data is available in the Zenodo repository, linked in the Supplementary Materials section.

## Supplementary Materials

The following supporting information can be downloaded at: https://www.mdpi.com/article/10.3390/v16050726/s1, Table S1: identified mutations; Table S2. Characteristics of participants included in the study; Figure S1. Heatmap of SNVs estimated frequencies; Figure S2. Geographic spread of the SNVs; Section S1. Primers design and sequences; Section S2. PCR conditions; Table S3 QC results of sequenced data; Table S4. Results of assembled sequences; Section S4. IGV inspection of identified novel mutations; Table S5: APOBEC3 derived mutations; Section S5. Sanger Sequencing analysis.
