# Supplementary material for: Exploring Viral Genome Profile in Mpox Patients during the 2022 Outbreak, in a North-Eastern Centre of Italy
Source: Viruses. 2024 May 3;16(5):726. doi: 10.3390/v16050726 (PMC11125733; doi:10.3390/v16050726)
Supplement: Supplementary file 1 [file viruses-16-00726-s001.zip › viruses-2983814-supplementary-revised.pdf]

Supplementary Material

Table S1: identified mutations. Available at the following link

Table S2. Characteristics of participants included in the study. Demographic information, collection time, qPCR Ct value, travel history, comorbidities, risk behaviors and the number of days until patients tested negative at qPCR.

ID	Sex	Age (years old)	Collection data	qPCR (Ct value)	Travel history	Immune system diseases	Analysed matrix	sexual risk behaviors	Positivity period (days)
**P1**	**M**	**44**	**June 2022**	**20.00**	**Yes, Palma de Mallorca**	**HIV**	**Lesion swab**	**Yes**	**22**
**P2**	**M**	**46**	**July 2022**	**18.90**	**Yes, Spain**	**HIV**	**Lesion swab**	**Yes**	**23**
**P3**	**M**	**43**	**October 2022**	**12.79**	**No**	**none**	**Lesion swab**	**Yes**	**21**
**P4**	**M**	**45**	**November 2022**	**16.02**	**Yes, Bosnia-Herzegovina**	**none**	**Lesion swab**	**Not available**	**34**

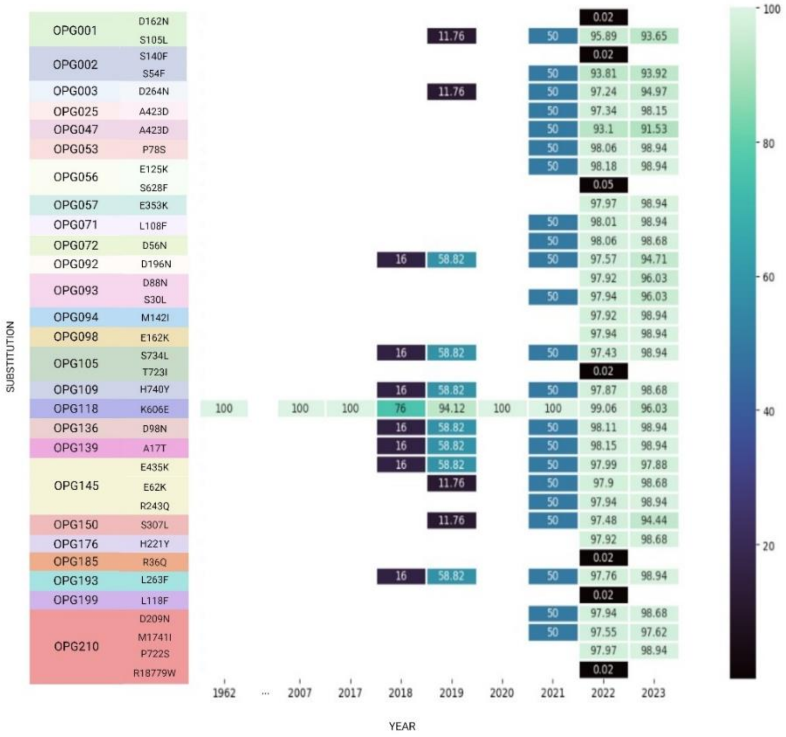

Figure S1. Heatmap showing estimated frequencies of the 36 mutations (known and novel) identified in the viral genome from samples of the four patients analysed in the present study. Each column represents a year. Variants are ordered by year of detection. Each row represents a single variant. Colours depict the mutation frequency in the database: the clearer colour indicating the higher mutation frequency. The heatmap was generated using the function heatmap from the Python data visualization library seaborn (https://seaborn.pydata.org/generated/seaborn.heatmap.html, last accessed 5 July 2023).

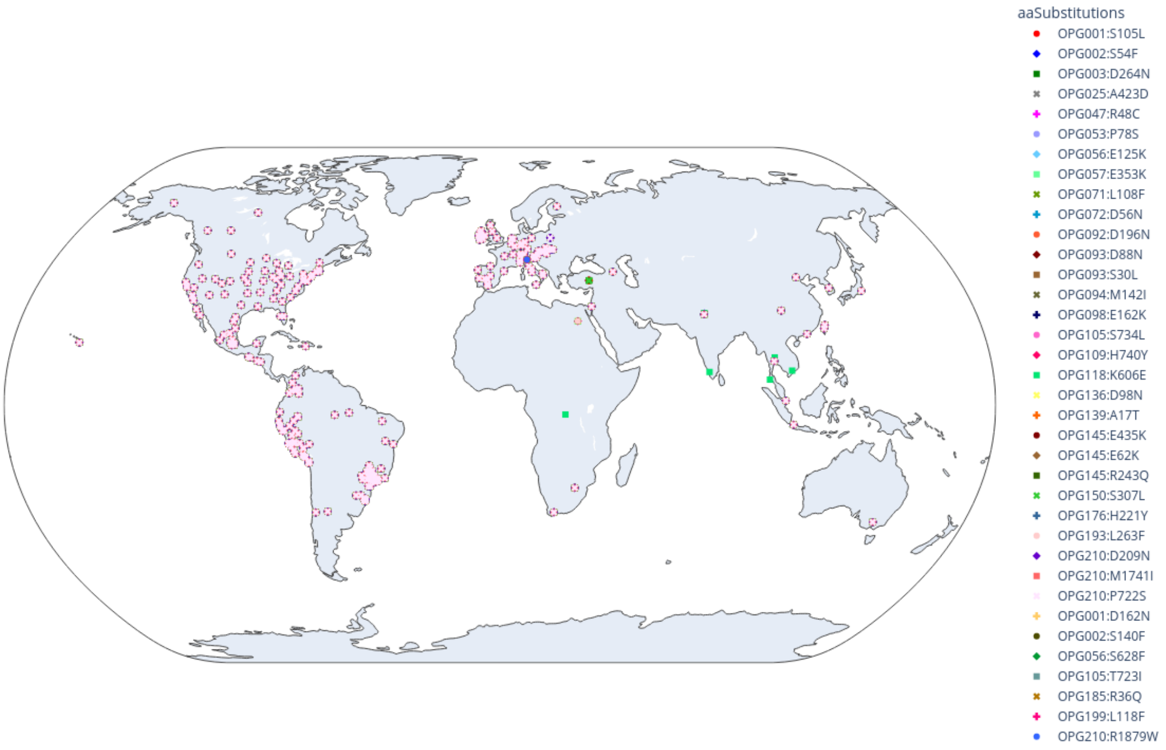

Figure S2. Geographic spread of the 37 SNVs.

Section S1. Primers design and sequences.

Primers were designed using OligoPerfect Primer Designer (ThermoFisher).

Gene name	SNV location	Amplicon size	Primer Name	Primer sequence
**OPG210**	186990	256 bp	OPG210_fw	TGGCAATTTATATAGCGCGCA
			OPG210_rv	CGGTAGTTACATATTACCATATCATCC
**OPG199**	171841	232 bp	OPG199_fw	GGCGATATTCTGCCGTGTTT
			OPG_199_rv	GGTTGATGCCATTCGAAAAGGA
**OPG185**	159023	202 bp	OPG185_fw	TGACACAATTACCAATACTTTTGTTAC
			OPG_185_rv	AAAAGCGACGTCTTGTATTTTGA

Section S2. PCR conditions

Component	Volume
**Q5 High-Fidelity 2X Master Mix**	12.5µl
**10 µM Forward Primer**	1.25µl
**10 µM Reverse Primer**	1.25 µl
**Nuclease-Free Water**	5 µl
**DNA**	5 µl
**TOTAL VOLUM**	25 µl

STEP	TEMPERATURE	TIME
**Initial Denaturation**	98°C	30 seconds
**35 Cycles**	98°C	8 seconds
	OPG210 59°C<br>OPG199 61°C<br>OPG185 58°C	30 seconds
	72°C	30 seconds
**Final Extension**	72°C	2 minutes
**Hold**	4°C	∞

Section S3. NGS results

Table S3 QC results of sequenced data. For each sample, the columns show the GISAID EPID (if available), the total number of sequenced fragments, the percentage of GC content, the number of mapped fragments on MPXV reference, the mean depth of coverage and the percentage of target sequences covered at least by 1/10/30 reads.

ID	EPID	Total n° of sequenced fragments	GC (%)	N° of fragments mapped on MPXV	Mean depth coverage (X)	1X (%)	10X (%)	30X (%)
**P1**	EPI_ISL_14786290	23,846,609	42	28,971	35.70	100	99.27	69.89
**P2**	EPI_ISL_14786346	17,467,127	41	57,273	71.59	100	99.93	99.72
**P4**	EPI_ISL_16467111	16,492,867	40	94,306	91.09	99.99	99.85	99.56
**P3**	-	2,572,696	39	19,017	13.68	99.90	79.98	12.11

Table S4. Results of assembled sequences. For each sample the columns show the GISAID EPID (if available), the total length of the genome assembled with iVar, the total number of unidentified nucleotides (Ns) present in the assembled genomes, and the number of gaps, identified SNVs, Indels, frameshifts, amino acid substitutions, deletions and insertions.

ID	EPID	Assembly length	Coverage (%)	# Ns	Gaps	# SNVs	# dels.	# ins.	# frameshift	# AA subs.	# AA dels	# AA ins.
**P1**	EPI_ISL_14786290	197,120	99.92 %,	1,501	34	68	116	15	10	30	8	0
**P2**	EPI_ISL_14786346	197,082	99.83 %	164	7	73	151	18	14	27	15	0
**P4**	EPI_ISL_16467111	197,197	99.24 %	343	13	74	13	1	0	33	0	0
**P3**	-	197,210	80.04 %	39,353	2	61	2	27	16	26	1	1

Section S4. IGV inspection of identified novel mutations

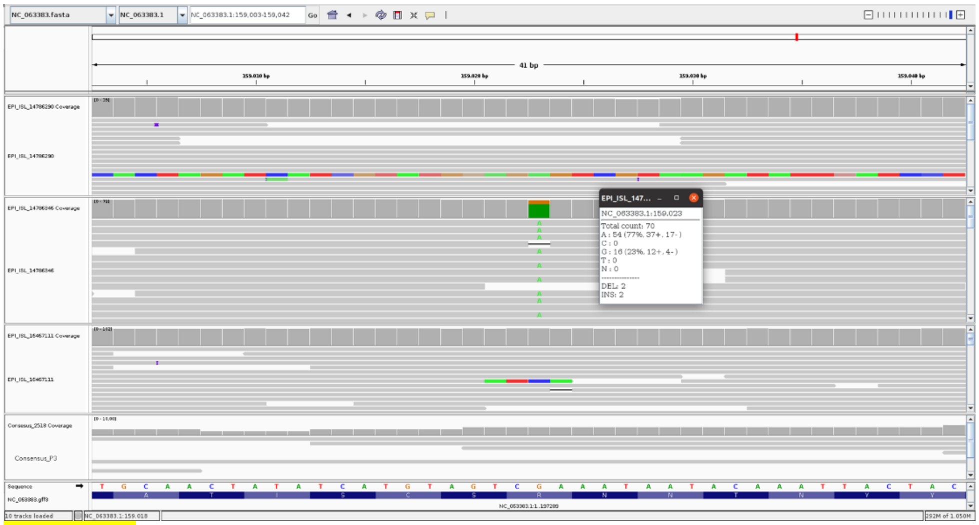

Figure S4 variants G159023A located on OPG185 gene

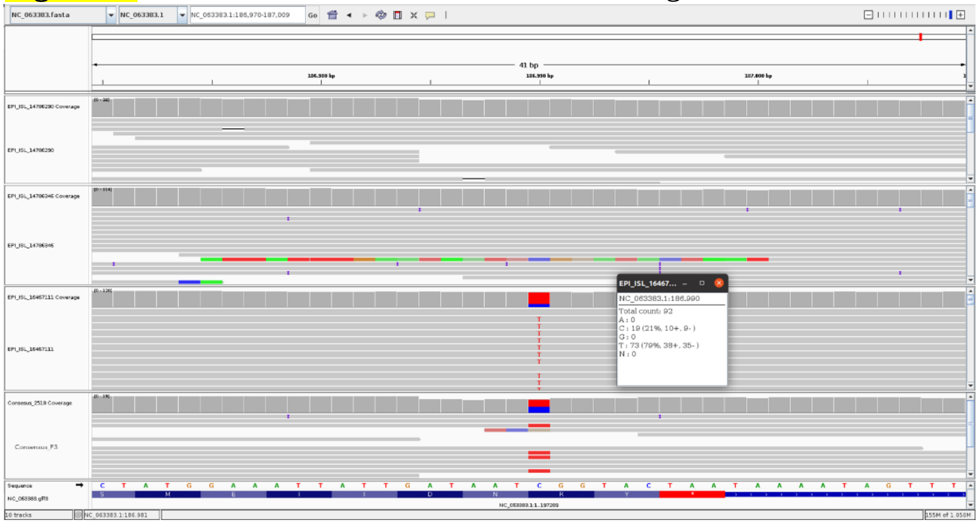

Figure S4.1 shows the variants C186990T located in the gene OPG210

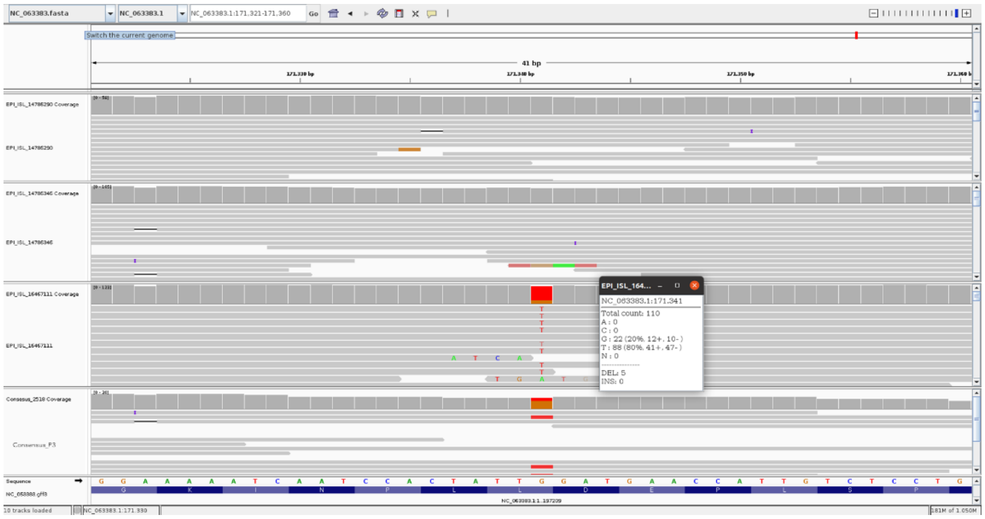

Figure S4.2 The variants G171341T located on OPG199 gene

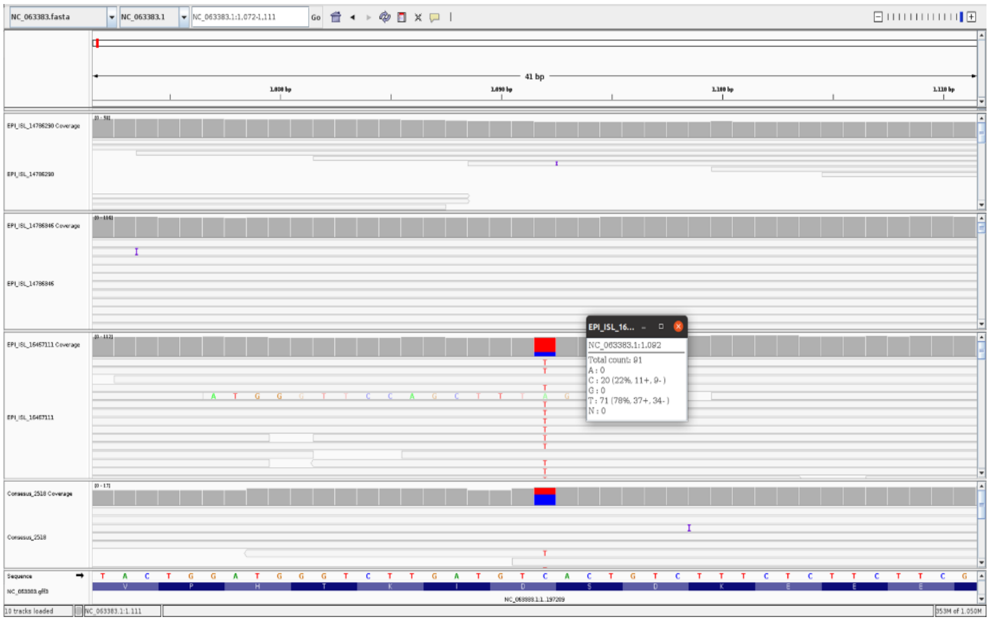

Figure S4.3 The variants C1092T located on OPG001 gene, called by white reads

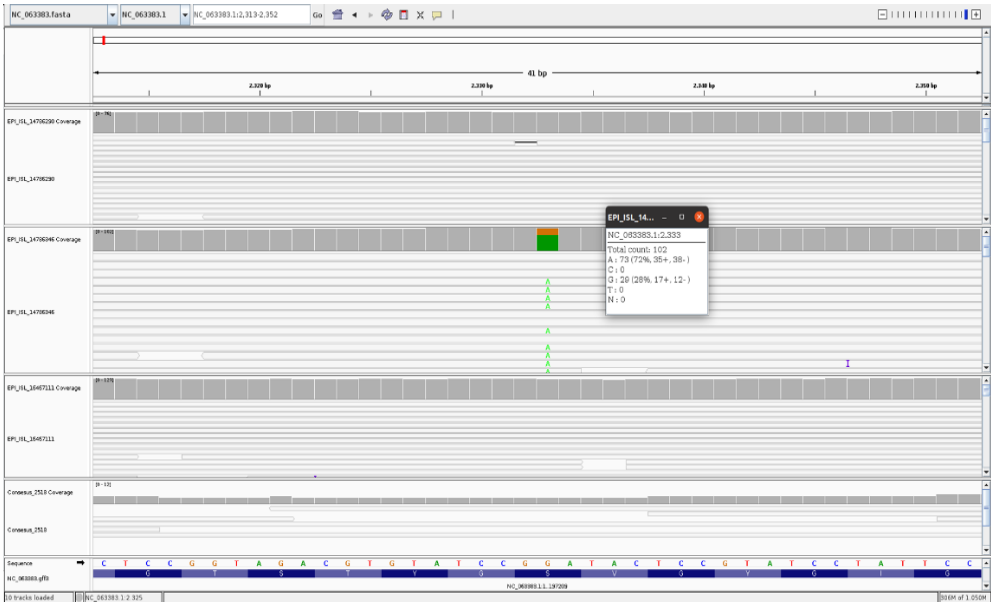

Figure S4.4 The variants G2333A located on OPG002 gene, called by white reads

Table S5: APOBEC3 derived mutations, available at the following link

Section S5. Sanger Sequencing analysis

Sample P2- mutation OPG185: R36Q - G159023A

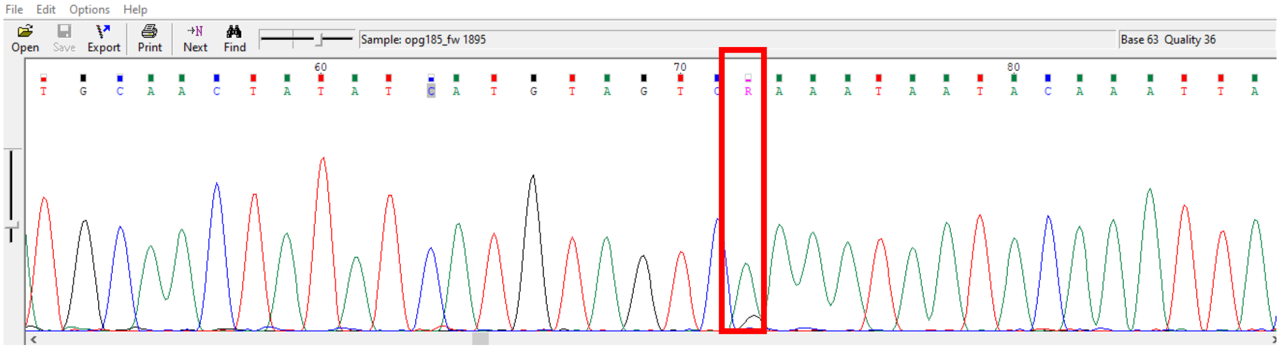

Download GenBank Graphics

Monkeypox virus isolate MPXV/Germany/2022/ON/RKI904, partial genome

Sequence ID: OQ773523.1 Length: **190625** Number of Matches: **1**

Range 1: 158896 to 159046 GenBank Graphics ▼ Next Match ▲ Previous Match

Score	Expect	Identities	Gaps	Strand
276 bits(149)	9e-70	150/151(99%)	0/151(0%)	Plus/Plus

```
Query  1       ACACCTTCTCCTCAGACATCTaaaaaaaTAGGTGATGATGCAACTATATCATGTAGTCRA  60
               |||||||||||||||||||||||||||||||||||||||||||||||||||||||||| |
Sbjct  158896  ACACCTTCTCCTCAGACATCTAAAAAAATAGGTGATGATGCAACTATATCATGTAGTCGA  158955

Query  61      AATAATACAAATTACTACGTTGTTATGAGTGCTTGGTATAAGGAGCCCAATTCCATTATT  120
               ||||||||||||||||||||||||||||||||||||||||||||||||||||||||||||
Sbjct  158956  AATAATACAAATTACTACGTTGTTATGAGTGCTTGGTATAAGGAGCCCAATTCCATTATT  159015

Query  121     CTCTTAGCTGCCAAAAGCGACGTCTTGTATT  151
               |||||||||||||||||||||||||||||||
Sbjct  159016  CTCTTAGCTGCCAAAAGCGACGTCTTGTATT  159046
```

Fare clic o toccare qui per immettere il testo.

Sample P3- mutation OPG210:R1879Q – C186990T

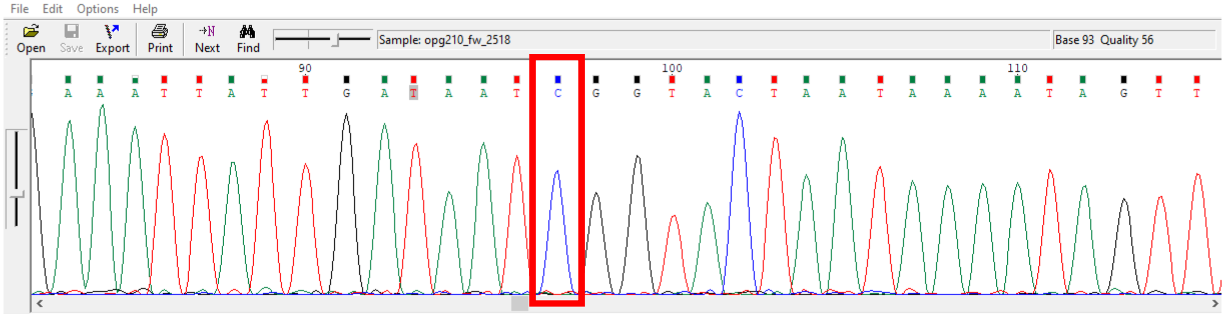

Download GenBank Graphics

Monkeypox virus isolate MPXV/Germany/2022/ON/RKI904, partial genome

Sequence ID: OQ773523.1 Length: **190625** Number of Matches: **1**

Range 1: 186794 to 186947 GenBank Graphics ▼ Next Match ▲ Previous Match

Score	Expect	Identities	Gaps	Strand
285 bits(154)	2e-72	154/154(100%)	0/154(0%)	Plus/Plus

```
Query  1       ATATGACAATATGAGCATTAAATCTGAGCATCATGATAGTCTTGAAACAGTGTCTATGGA  60
               ||||||||||||||||||||||||||||||||||||||||||||||||||||||||||||
Sbjct  186794  ATATGACAATATGAGCATTAAATCTGAGCATCATGATAGTCTTGAAACAGTGTCTATGGA  186853

Query  61      AATTATTGATAATCGGTACTAATAAAATAGTTTAACTCTTTTAGAACCAGTTTGGTACTG  120
               ||||||||||||||||||||||||||||||||||||||||||||||||||||||||||||
Sbjct  186854  AATTATTGATAATCGGTACTAATAAAATAGTTTAACTCTTTTAGAACCAGTTTGGTACTG  186913

Query  121     TAATTTCAGTTCATTACTCGTTGAGAATATTGAT  154
               ||||||||||||||||||||||||||||||||||
Sbjct  186914  TAATTTCAGTTCATTACTCGTTGAGAATATTGAT  186947
```

Sample P3- mutation OPG199:L118F – G171841T

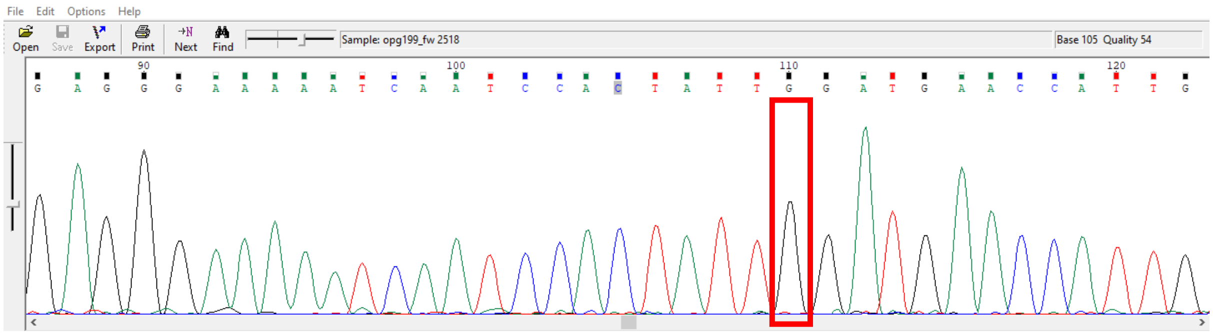

Download GenBank Graphics

Monkeypox virus isolate MPXV/Germany/2022/ON/RKI904, partial genome

Sequence ID: OQ773523.1 Length: 190625 Number of Matches: 1

Range 1: 171186 to 171305 GenBank Graphics ▼ Next Match ▲ Previous M

Score	Expect	Identities	Gaps	Strand
222 bits(120)	9e-54	120/120(100%)	0/120(0%)	Plus/Plus

```
Query  1       ACTGTTGACTTCACTGATTGTCGCACTATAGATGCAATCAATAAGTGTGTAGATATCTTT  60
               ||||||||||||||||||||||||||||||||||||||||||||||||||||||||||||
Sbjct  171186  ACTGTTGACTTCACTGATTGTCGCACTATAGATGCAATCAATAAGTGTGTAGATATCTTT  171245

Query  61      ACTGAGGGAAAAATCAATCCACTATTGGATGAACCATTGTCTCCTGATACCTGTCTCCTA  120
               ||||||||||||||||||||||||||||||||||||||||||||||||||||||||||||
Sbjct  171246  ACTGAGGGAAAAATCAATCCACTATTGGATGAACCATTGTCTCCTGATACCTGTCTCCTA  171305
```

Sample P4 - mutation OPG210:R1879Q – C186990T

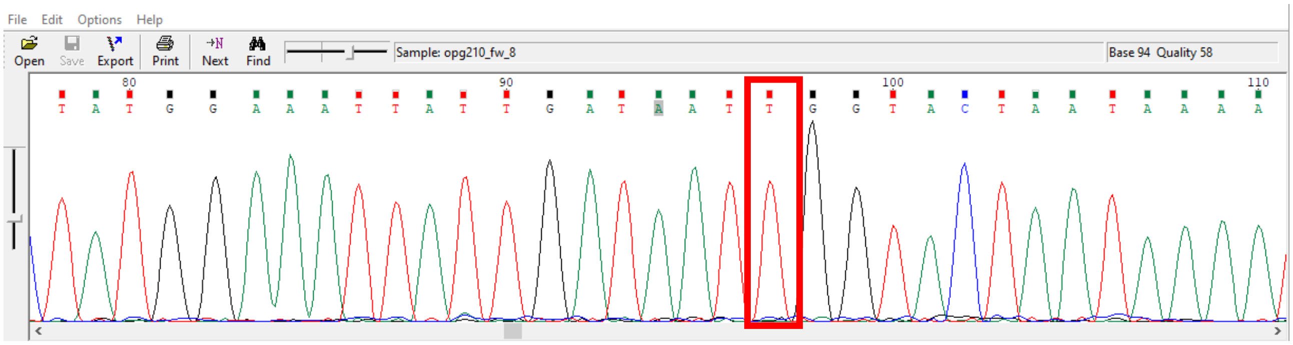

Download GenBank Graphics

Monkeypox virus isolate MPXV/Germany/2022/ON/RKI904, partial genome

Sequence ID: OQ773523.1 Length: 190625 Number of Matches: 1

Range 1: 186786 to 186934 GenBank Graphics ▼ Next Match ▲ Previous Match

Score	Expect	Identities	Gaps	Strand
270 bits(146)	4e-68	148/149(99%)	0/149(0%)	Plus/Plus

```
Query  1       ATAATGAAATATGACAATATGAGCATTAAATCTGAGCATCATGATAGTCTTGAAACAGTG  60
               ||||||||||||||||||||||||||||||||||||||||||||||||||||||||||||
Sbjct  186786  ATAATGAAATATGACAATATGAGCATTAAATCTGAGCATCATGATAGTCTTGAAACAGTG  186845

Query  61      TCTATGGAAATTATTGATAATTGGTACTAATAAAATAGTTTAACTCTTTTAGAACCAGTT  120
               ||||||||||||||||||||| ||||||||||||||||||||||||||||||||||||||
Sbjct  186846  TCTATGGAAATTATTGATAATCGGTACTAATAAAATAGTTTAACTCTTTTAGAACCAGTT  186905

Query  121     TGGTACTGTAATTTCAGTTCATTACTCGT  149
               |||||||||||||||||||||||||||||
Sbjct  186906  TGGTACTGTAATTTCAGTTCATTACTCGT  186934
```

Sample P4 mutation OPG199:L118F – G171841T

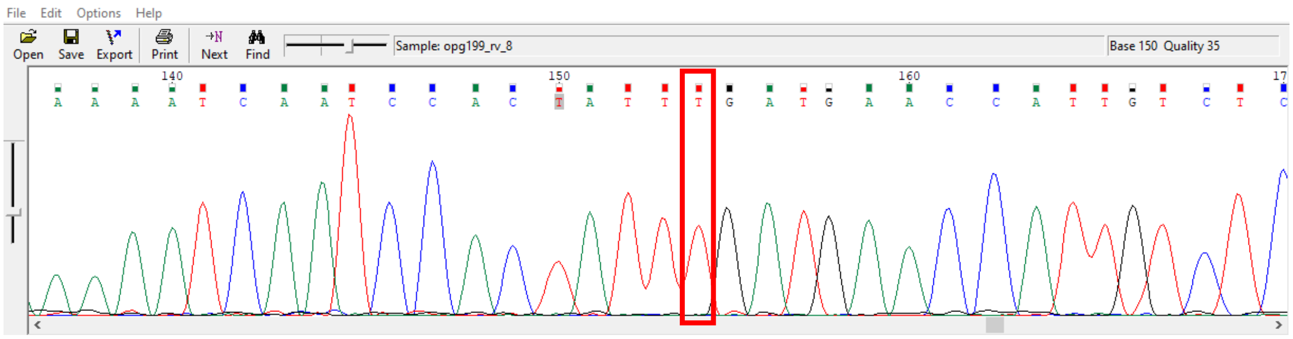

Download GenBank Graphics

Monkeypox virus isolate MPXV/Germany/2022/ON/RKI904, partial genome

Sequence ID: OQ773523.1 Length: 190625 Number of Matches: 1

Range 1: 171135 to 171307 GenBank Graphics ▼ Next Match ▲ Previous

Score	Expect	Identities	Gaps	Strand
303 bits(164)	5e-78	169/173(98%)	0/173(0%)	Plus/Plus

```
Query  1       TCTGCCGTGTTTAAAGATTCCTTTTTGGGAAAAATTGGCGATAAGTTTYWWACTGTTGAC  60
               ||||||||||||||||||||||||||||||||||||||||||||||||   |||||||||
Sbjct  171135  TCTGCCGTGTTTAAAGATTCCTTTTTGGGAAAAATTGGCGATAAGTTTCAAACTGTTGAC  171194

Query  61      TTCACTGATTGTCGCACTATAGATGCAATCAATAAGTGTGTAGATATCTTTACTGAGGGA  120
               ||||||||||||||||||||||||||||||||||||||||||||||||||||||||||||
Sbjct  171195  TTCACTGATTGTCGCACTATAGATGCAATCAATAAGTGTGTAGATATCTTTACTGAGGGA  171254

Query  121     AAAATCAATCCACTATTTGATGAACCATTGTCTCCTGATACCTGTCTCCTAGC  173
               ||||||||||||||||| |||||||||||||||||||||||||||||||||||
Sbjct  171255  AAAATCAATCCACTATTGGATGAACCATTGTCTCCTGATACCTGTCTCCTAGC  171307
```
